# Dispersion in porous media in oscillatory flow between flat plates: applications to intrathecal, periarterial and paraarterial solute transport in the central nervous system

**DOI:** 10.1186/s12987-019-0132-y

**Published:** 2019-05-06

**Authors:** M. Keith Sharp, Roxana O. Carare, Bryn A. Martin

**Affiliations:** 1 Biofluid Mechanics Laboratory, Department of Mechanical Engineering, University of Louisville, Louisville, KY 40292 USA; 2 Faculty of Medicine, Southampton General Hospital, University of Southampton, Southampton, SO16 6YD UK; 3 Department of Biological Engineering, University of Idaho, Moscow, ID 83844 USA

**Keywords:** Perivascular flow, Paravascular flow, Paravenous flow, Spinal subarachnoid space, Cerebrospinal fluid, Glymphatic system

## Abstract

**Background:**

As an alternative to advection, solute transport by shear-augmented dispersion within oscillatory cerebrospinal fluid flow was investigated in small channels representing the basement membranes located between cerebral arterial smooth muscle cells, the paraarterial space surrounding the vessel wall and in large channels modeling the spinal subarachnoid space (SSS).

**Methods:**

Geometries were modeled as two-dimensional. Fully developed flows in the channels were modeled by the Darcy–Brinkman momentum equation and dispersion by the passive transport equation. Scaling of the enhancement of axial dispersion relative to molecular diffusion was developed for regimes of flow including quasi-steady, porous and unsteady, and for regimes of dispersion including diffusive and unsteady.

**Results:**

Maximum enhancement occurs when the characteristic time for lateral dispersion is matched to the cycle period. The Darcy–Brinkman model represents the porous media as a continuous flow resistance, and also imposes no-slip boundary conditions at the walls of the channel. Consequently, predicted dispersion is always reduced relative to that of a channel without porous media, except when the flow and dispersion are both unsteady.

**Discussion/conclusions:**

In the basement membranes, flow and dispersion are both quasi-steady and enhancement of dispersion is small even if lateral dispersion is reduced by the porous media to achieve maximum enhancement. In the paraarterial space, maximum enhancement *R*_*max*_ = 73,200 has the potential to be significant. In the SSS, the dispersion is unsteady and the flow is in the transition zone between porous and unsteady. Enhancement is 5.8 times that of molecular diffusion, and grows to a maximum of 1.6E+6 when lateral dispersion is increased. The maximum enhancement produces rostral transport time in agreement with experiments.

**Electronic supplementary material:**

The online version of this article (10.1186/s12987-019-0132-y) contains supplementary material, which is available to authorized users.

## Introduction

### Motivation

An attractive avenue for drug transport to the brain is the spinal subarachnoid space (SSS). Inconsistent results suggest that more complete understanding of solute dispersion in the SSS could improve outcomes. Similarly, solute transport in the so-called “glymphatic system” has been observed and has been hypothesized to be an important route for clearing metabolites and regulating immune response, but controversy exists over the mechanisms of the transport, and even of the existence of net flow in the perivascular spaces. A phenomenological feature that these two spaces potentially have in common is the presence of oscillatory flow (zero net flow component). Oscillatory flow offers the possibility that at least a portion of the observed solute transport may be due to shear-augmented (Taylor) dispersion, rather than bulk flow. This paper uses a mathematical model and order-of-magnitude estimates to evaluate the plausibility of significant Taylor dispersion in the SSS and “glymphatic system” spaces and the potential that conditions within the spaces might be clinically controlled to optimize transport.

The remainder of this “Introduction” section will first describe Taylor dispersion (in “Shear-augmented dispersion” section) and then summarize the relatively well-known anatomy and flow and transport parameters of the SSS (see “Intrathecal flow and transport” section), and the same, but so far incompletely understood, parameters for the paravascular and perivascular spaces (see “Perivascular and paravascular flow and transport” section).

### Shear-augmented dispersion

Axial transport of solutes can be reduced or enhanced by diffusion across streamlines. For example, in steady, purely axial pipe flow, a bolus of a passive species is carried forward faster in the center of the pipe than near the walls, creating radial concentration gradients that favor diffusion toward the walls of the pipe at the leading edge of the bolus and toward the center of the pipe at the trailing edge. The spread of the bolus is, therefore, reduced by diffusion from high-velocity to low-velocity streamlines on the leading edge, and by diffusion from low- to high-velocity streamlines on the trailing edge (called Taylor dispersion in honor of Taylor [1]). In oscillatory (fluctuating with zero mean), purely axial flow, net axial transport is zero in the absence of diffusion. Transverse diffusion similar to the steady case increases axial dispersion by leaving some of the tracer behind on streamlines of lower velocity as the flow reverses after having been carried forward on high-velocity streamlines [2]. Transverse convection can also spread the tracer across axial streamlines of different velocities, for instance, by secondary flows in a curved pipe [3]. When the time constants for axial displacement and transverse mixing are matched, the augmentation *R* of axial dispersion relative to molecular diffusion is greatly enhanced, analogous to tiny delivery vehicles hauling tracer forward and returning empty with each displacement cycle [3, 4].

### Perivascular and paravascular flow and transport

**Fig. 1:**
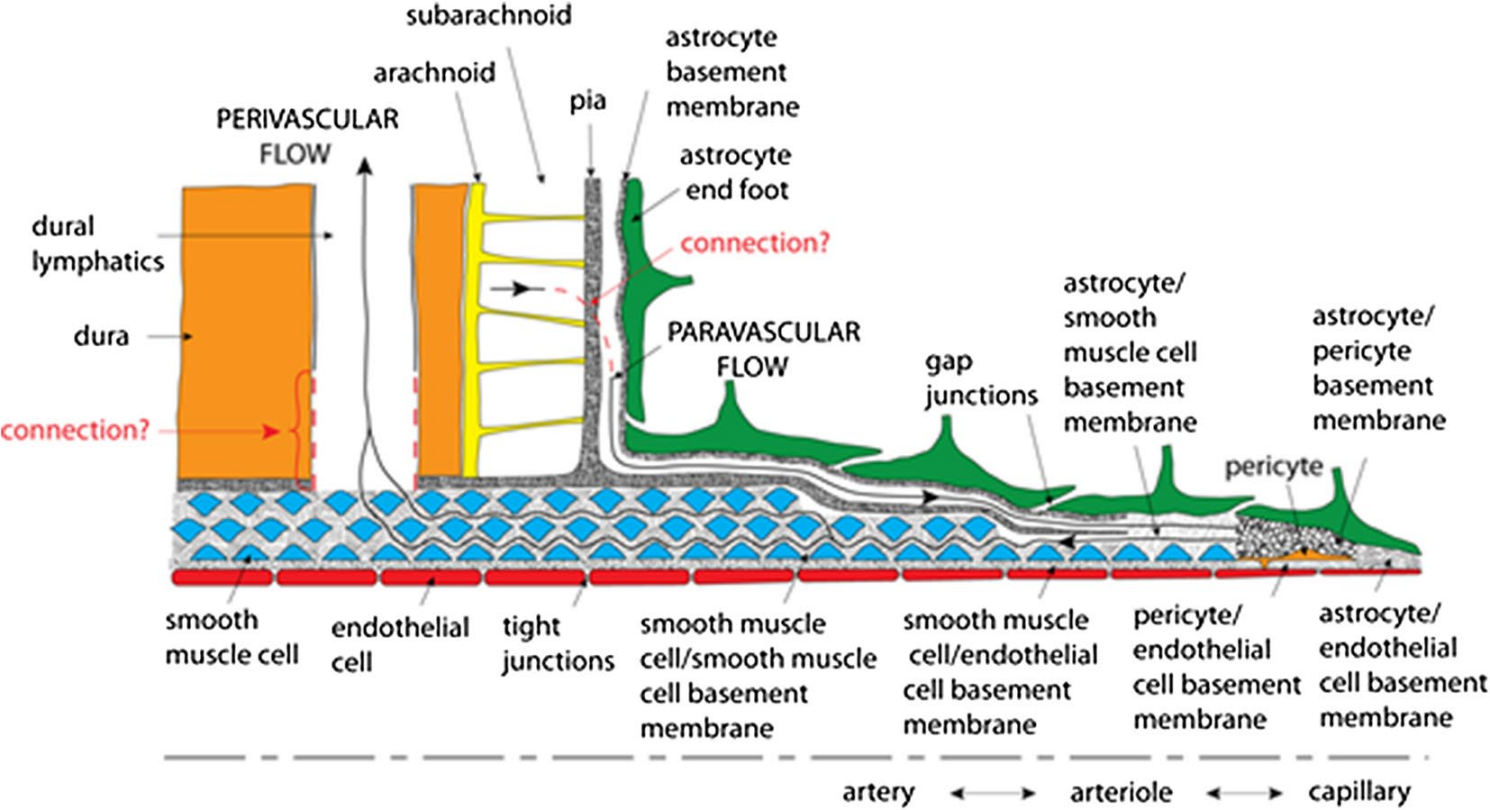
Hypothetical perivascular and paravascular flow pathways in an artery. Paravascular flow is hypothesized to move inward to the brain tissue between astrocyte end feet and pia mater. Perivascular flow is hypothesized to move outward from the brain tissue in basement membranes between smooth muscle cells. (From [33])

Historically, when only the Virchow-Robin space (VRS) was recognized, this space was called perivascular. However, as the potential was found for transport in two different channels around cerebral blood vessels (Fig. 1), a different nomenclature has been adopted. First, perivascular refers to the space within the wall of a cerebral artery, specifically in the basement membranes (about 100 nm thickness) between smooth muscle cells (SMC), which form rings about 2–6 μm wide that wrap around the circumference of the vessel by about 1.5 turns [5, 6]. One layer of SMCs is present in the circumference of the arterioles, while 4–20 layers are found in larger arteries [6]. Observations on human brains with cerebral amyloid angiopathy and experimental studies using tracers injected into the parenchyma suggests that interstitial fluid (ISF) flows out of the brain tissue via the intramural periarterial drainage (IPAD) pathways in the direction opposite that of blood flow within the artery (Fig. 1). This direction of IPAD is inferred based on tracers of various sizes that were injected into the brain parenchyma and found in the basement membranes between SMC’s, but not in the 30–40 nm thick basal lamina between endothelial cells and SMC’s, nor in the basement membrane outside the outermost layer of SMC’s [7]. Identifying a mechanism for retrograde flow is key to validating the IPAD concept (e.g., [8–10]). The tracers eventually drain to cervical lymph nodes [11–13]. Failure of this process with increasing age and with risk factors for Alzheimer’s disease may lead to the accumulation of proteins in the walls of arteries, but not veins, as observed in human cases and animal models of cerebral amyloid angiopathy [14, 15].

Second, paravascular flow is hypothesized to occur outside the vessel wall, i.e., outside the outermost SMCs, but enclosed within the astrocyte end feet forming the glia limitans (Fig. 1). Convective influx of cerebrospinal fluid (CSF) is thought to occur from the cortical subarachnoid space (CSS) along these paraarterial spaces to combine with ISF as it flows into the parenchyma near the capillaries [16, 17]. According to the glymphatic hypothesis, ISF is cleared along similar paravenous channels back to the CSS. The paraarterial space has been considered synonymous with the Virchow-Robin space (VRS) without a clear description of the anatomical structures that form its boundaries [16, 18]. Historically, it was speculated that the VRS was bounded on the outside by the pia and freely communicated with CSF in the CSS [19, 20]. However, electron microscopy revealed that the pial sheath is closely associated with the abluminal part of SMC’s and blocks such circulation by covering arteries both upstream and downstream of the pia mater surrounding the brain (see Fig. 1) [21, 22]. Therefore, the inner wall of this pathway may be the pia. VRS between the pia and glia limitans is found in normal subjects when MRI sequences conducive to its detection are used [23]. The VRS is therefore a potential space formed between the glia limitans and the pial sheath, enlarging in ageing and cerebral amyloid angiopathy, possibly reflecting excess fluid that is unable to be cleared efficiently. A large, empty VRS, as traditionally envisioned (Fig. 1), is not universally presented. In these studies, the pia mater and glia limitans were separated only by their respective basement membranes [24–26]. Further, large paraarterial channels may be an artifact of high tracer infusion rates that inflate the space [13, 27]. On the other hand, fixation has been observed to reduce the paravascular cross sectional area by a factor of 10 [28]. Rather than judge which channel characteristics are most physiologically accurate, this paper will analyze both, with thin pial-glial basement membranes being addressed by the periarterial model, and thicker VRS channels by the paraarterial model.

The intriguing potential exists for simultaneous flows in opposite directions within the two different channels [29]. It should also be noted that the pial sheath is not found around veins in the parenchyma [22] which has implications for outflow along veins, as proposed as a part of the glymphatic circulation [16]. This outflow, if it exists, would have to occur in a different space, for instance, the collagen layer between the endothelium and the glia limitans [22].

While numerous experiments have documented transport of solutes within these spaces [12, 16], bulk flow of fluids has been directly verified only around the middle cerebral artery (MCA), in large part due to the difficulty of real-time measurements in the extremely small channels. Around the MCA, a mean velocity of 18.7 μm/s was measured by particle tracking [28]. However, this velocity corresponds to a flow rate of about 0.00308 μL/min that followed an infusion of tracer into the cisterna magna of 2 μL/min. The question is raised whether the relatively large infusion (about 2% of brain volume) inflated the cistern and caused the roughly 1000-fold smaller flow. The mechanism by which bulk flow may be driven has not been identified, but was thought to be related to the blood pressure pulse, because transport ceases after the heart is stopped in mice [12]. However, more recent modeling has shown that the stiffness of the middle cerebral artery is too large to allow significant flow to be driven by arterial wall motion [30]. The mean pressure difference between CSF and the central nervous system (CNS) parenchyma is small, about 1 mmHg or less [31, 32]. Therefore, its contribution to bulk flow may be insignificant. Further, the resistance of the cerebral paraarterial tree is too great to support bulk flow [33]. In this paper, an alternative hypothesis is evaluated that solute transport may occur in the absence of net bulk flow by shear-augmented dispersion.

### Intrathecal flow and transport

**Fig. 2:**
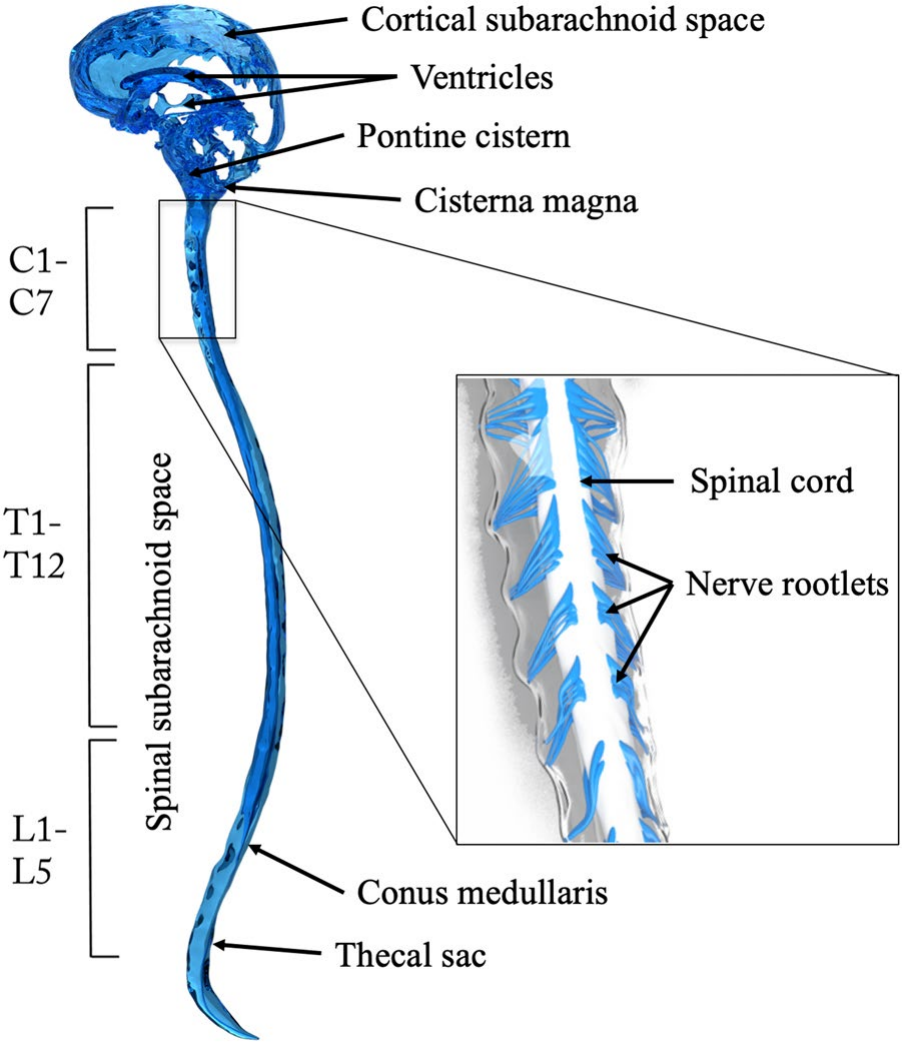
Anatomic diagram of the CSF system including spinal subarachnoid space (SSS) and cortical subarachnoid space (CSS) with ventricles and cisterns of the brain

**Fig. 3:**
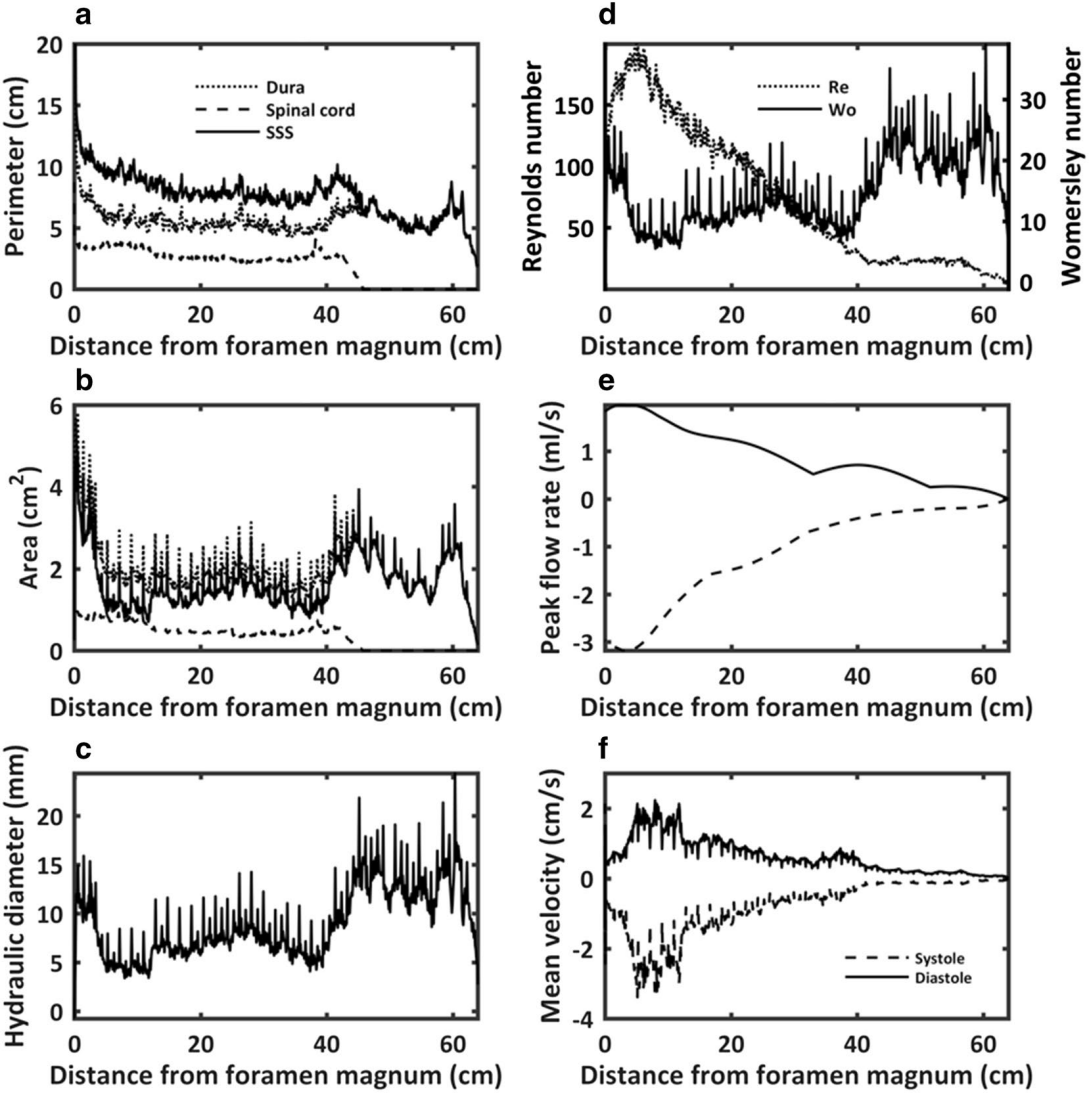
Example of geometric and hydrodynamic characterization of the SSS for a healthy adult male subject based on subject specific MRI measurements and engineering post-processing techniques described by Sass et al. [35]. Axial distribution of dura, spinal cord and SSS (dura + spinal cord) perimeter (**a**), dura, spinal cord and SSS area (**b**), hydraulic diameter (**c**), Reynolds and Womersley number (**d**), peak CSF flow rate at systole and diastole (**e**), mean CSF flow velocity at systole and diastole (**f**). Systolic flow is directed towards the feet

CSF pulsates with each cardiac cycle around the brain and spinal cord with nearly zero net flow. Features of the CSF system anatomy (Fig. 2) and physiology were reviewed by Martin et al. [34]. Total CSF volume ranges from 250 to 400 mL in an adult human [35] with ~ 90 mL located in the SSS. CSF is a clear fluid having similar properties as water at body temperature with density, *ρ* = 993 kg/m^3^ and kinematic viscosity, *ν* = 7 × 10^−7^ m^2^/s at body temperature [36]. Figure 3 indicates hydrodynamic and geometric characterization of the SSS for a healthy adult male subject in terms of key parameters. Computational fluid dynamics modeling of CSF flow has estimated Reynolds number based on hydraulic diameter to be from 150 to 450 within the SSS [37] and 340 within the aqueduct of Sylvius [38], which are both in the laminar range. Studies have indicated that jets and possible flow instabilities may be present [39]. The Womersley number^1^ in the SSS has been estimated to range from ~ 5 to 15 [40], which is unsteady.

The SSS can be considered to be a porous medium as described previously by Gupta et al. [41] and others. This is because the SSS is bounded by the pia-arachnoid complex [42], a fluid space that contains numerous microscopic structures including arachnoid trabeculae, arachnoid “sheets” with holes [43], and blood vessels. The porosity of the human SSS is not known precisely. Thus, our approach estimated a range of plausible values based on known anatomic dimensions.

Since CSF pulsates around the entire brain and spine, it can be leveraged as a conduit to deliver therapies to the brain and spinal cord. While CSF-based delivery of drugs and biologics to the CNS is promising, there is relatively little information about the physics of CSF flow and solute transport, which has, in turn, slowed therapeutic development. At present, targeting and optimizing the delivery of these therapies is problematic because virtually nothing is known about CSF dynamics in many CNS diseases. A better understanding of CSF flow and transport could help to optimize delivery parameters and/or system design to ensure that the drug reaches targeted CNS tissue regions [44]. This was accented in a recent study that concluded, “Assessment of biomarkers that report the kinetics of CSF flux in prospective gene therapy patients might inform variable treatment outcomes and guide future clinical trial design” [45].

To the extent that flows through the ultrastructures within the spinal subarachnoid space and in the perivascular and paravascular channels may be driven by oscillatory pressure gradients, and that longitudinal transport may be enhanced by the resulting velocity gradients, a mathematical model is developed to quantify the enhancement.

### Objectives

The plausibility of significant shear-augmented dispersion in the SSS and in the paravascular and perivascular spaces will be evaluated by two methods. First, an analytical model of transport in oscillatory flow through a simplified channel filled with (Darcy–Brinkman) porous media representing the CNS spaces is used to calculate a low estimate of the enhancement of dispersion. Model results are presented over a wide range of parameters, as well as for parameter sets for each space that yield the largest plausible enhancement with the Darcy–Brinkman model, which neglects the transverse mixing that can occur in porous media. Second, order-of-magnitude analysis is used to estimate the maximum enhancement associated with a match between the transverse mixing time and the cycle period of the oscillatory flow. Together, these lower and upper bounds test whether Taylor dispersion may be significant in these spaces and demonstrate the potential for improvement in transport by clinical manipulation of the parameters.

## Methods

### Mathematical model

Flows in the channels are simplified to be that between flat plates. (Validity of this and other simplifications are discussed in “Values of parameters” section). No-slip and no-flux boundary conditions are applied at the walls. The Darcy–Brinkman model is used to approximate the resistance to flow of the structures within the channels. This model smooths the local heterogeneities of flow through the porous material to a purely axial superficial velocity, which is the mean velocity of a hypothetical continuum fluid filling the channel. This approximation allows an analytical solution, but has potential implications for transport that are estimated by order-of-magnitude analysis in “Regimes of dispersion” section. For these conditions, the dimensional unsteady Darcy–Brinkman equation describes the fluid flow

1 $$\frac{{\partial \tilde{u}_{s} }}{{\partial \tilde{t}}} = - \frac{1}{\rho }\frac{{\partial \tilde{p}}}{{\partial \tilde{x}}} + \nu_{e} \frac{{\partial^{2} \tilde{u}_{s} }}{{\partial \tilde{y}^{2} }} - \frac{\nu }{k}\tilde{u}_{s} ,$$

where *k* is permeability, $\tilde{p}$ is pressure, $\tilde{t}$ is time, $\tilde{u}_{s}$ is superficial axial velocity, $\tilde{x}$ is the axial coordinate, $\tilde{y}$ is the transverse coordinate, *ν* is the kinematic viscosity of the fluid, *ν*_*e*_ is the effective kinematic viscosity for flow in the porous medium, and *ρ* is the fluid density. The last term on the right-hand side, called the Darcy term, is an addition compared to the Navier–Stokes equation for flow without porous media. This term is significant for porous flow. $k \to \infty$ and $\nu_{e} \to \nu$ for nonporous flow.

Equation 1 is nondimensionalized as

2 $$\alpha^{2} \frac{\partial u}{\partial t} = - \frac{\partial p}{\partial x} + \frac{{\partial^{2} u}}{{\partial y^{2} }} - Da^{2} u,$$

where $p = \frac{{\tilde{p}}}{{\rho \omega \nu_{e} }}$ is pressure, *ω* is frequency, $t = \omega \tilde{t}$ is time, $u = \tilde{u}_{s} /h\omega$ is the superficial velocity, $x = \tilde{x}/h$ is the axial coordinate, $y = \tilde{y}/h$ is the transverse coordinate, *h* is the channel half height, $\alpha^{2} = \frac{{h^{2} \omega }}{{\nu_{e} }}$ is the square of the Stokes (Womersley) number and $Da^{2} = \frac{{h^{2} \nu }}{{k\nu_{e} }}$ is the square of the Darcy number ($Da \to 0$ for nonporous flow [2]).

Inserting a complex oscillatory pressure gradient $\frac{\partial p}{\partial x} = - Pe^{it}$, where $P = \frac{{\partial \tilde{p}/\partial \tilde{x}}}{{\rho \omega \nu_{e} /h}}$, the oscillatory velocity can be described as the real component of separable spatial and temporal parts $u = \text{Re} \left[ {f\left( y \right)e^{it} } \right]$. By inserting these pressure and velocity relationships into Eq. 2, the spatial part of the equation of motion is

3 $$\nabla^{2} f - d^{2} f = - P,$$

where $d^{2} \equiv M + iN = Da^{2} + i\alpha^{2}$ and the real and imaginary parts *m* and *n* of *d* are defined by $d \equiv m + in = \frac{1}{\sqrt 2 }\sqrt {\sqrt {Da^{4} + \alpha^{4} } + Da^{2} } + i\frac{1}{\sqrt 2 }\sqrt {\sqrt {Da^{4} + \alpha^{4} } - Da^{2} }$. (Note that $d^{2} = i\alpha^{2}$ for nonporous flow [2]). Equation 3 has the solution

4 $$f = \frac{P}{{d^{2} }}\left( {1 - F} \right),$$

where

5 $$F = \frac{\cosh dy}{\cosh d}.$$

Dimensional longitudinal dispersion is described by

6 $$\frac{\partial c}{{\partial \tilde{t}}} + \tilde{u}_{s} \frac{\partial c}{{\partial \tilde{x}}} = \kappa \tilde{\nabla }^{2} c,$$

where *c* is concentration of a passive tracer and *κ* is its molecular diffusivity, which can be nondimensionalized as

7 $$\nabla^{2} \theta - \beta^{2} \frac{\partial \theta }{\partial t} = \beta^{2} u\frac{\partial \theta }{\partial x},$$

where $\theta = \frac{c}{{c_{0} }}$, where *c*_*0*_ is a characteristic concentration, $\beta^{2} = \frac{{h^{2} \omega }}{\kappa } = \alpha^{2} Sc$ is the oscillatory Peclet number (hereafter simplified to the Peclet number) and $Sc = \nu /\kappa$ is the Schmidt number. Equation 7 is the same as the nonporous case [2], but *u* is now a function of *Da*, which leads to a *Da* dependence for *θ.*

From Eqs. 2 & 7, dimensional analysis reduces the number of variables to

8 $$u,\theta = u,\theta \left( {P,t,x,y,\alpha ,Da,Sc} \right).$$

Inserting the velocity solution *f* and a separable concentration profile $\theta = - \gamma x + \text{Re} \left[ {\gamma g\left( y \right)e^{it} } \right]$ that includes an oscillatory component that is independent of axial location and steady state longitudinal concentration gradient that is uniform across the cross section $\gamma = - \partial \theta /\partial x = const$, gives

9 $$\nabla^{2} g - i\beta^{2} g = - \beta^{2} f,$$

which has the solution

10 $$g = A + B\cosh dy + C\cosh ry,$$

where $A = \frac{P}{{d^{2} i}}$, $B = \frac{{P\beta^{2} }}{{d^{2} \left( {d^{2} - r^{2} } \right)\cosh d}}$, $C = - \frac{Bd\sinh d}{r\sinh r}$, $r^{2} = \frac{{ih^{2} \omega }}{\kappa } = i\beta^{2}$, $r = \sqrt {i\beta^{2} } = \bar{r}\left( {1 + i} \right)$ and $\bar{r} = \beta /\sqrt 2$. The flux of tracer per unit depth is

11 $$\tilde{j} = \int_{0}^{h} {\left( {\tilde{u}c - \kappa \frac{\partial c}{{\partial \tilde{x}}}} \right)} d\tilde{y},$$

which in dimensionless form becomes

12 $$j \equiv \frac{{\tilde{j}}}{h\omega } = \int_{0}^{1} {\left( {u\theta - \frac{\kappa }{{h^{2} \omega }}\frac{\partial \theta }{\partial x}} \right)} dy = \int_{0}^{1} {u\theta } dy + \frac{\gamma }{{\beta^{2} }}.$$

Using complex conjugates (designated by an overbar), velocity becomes $u = \text{Re} \left[ {f\left( y \right)e^{it} } \right] = \frac{1}{2}\left( {fe^{it} + \bar{f}e^{ - it} } \right)$ and concentration $\theta = - \gamma x + \text{Re} \left[ {\gamma g\left( y \right)e^{it} } \right] = - \gamma x + \frac{\gamma }{2}\left( {ge^{it} + \bar{g}e^{ - it} } \right)$.

The product of velocity and concentration is then $u\theta = \frac{1}{2}\left( {fe^{it} + \bar{f}e^{ - it} } \right)\left[ { - \gamma x + \frac{\gamma }{2}\left( {ge^{it} + \bar{g}e^{ - it} } \right)} \right] = - \frac{\gamma x}{2}\left( {fe^{it} + \bar{f}e^{ - it} } \right) + \frac{\gamma }{4}\left( {fge^{i2t} + f\bar{g}e^{0} + \bar{f}ge^{0} + \bar{f}\bar{g}e^{i2t} } \right)$.

Neglecting the oscillatory terms in the product, which do not contribute to flux over times long compared to the oscillatory period, the flux becomes

13 $$j = \frac{\gamma }{4}\int_{0}^{1} {\left( {f\bar{g} + \bar{f}g} \right)} dy + \frac{\gamma }{{\beta^{2} }}.$$

The effective diffusivity is defined (following Watson [2]) as

14 $$D_{eff} \equiv \frac{{\tilde{j}}}{\partial c/\partial x} = \kappa \left( {1 + R} \right),$$

where the enhancement of transport by shear is

15 $$R = \frac{1}{4}\int\limits_{0}^{1} {\left( {f\bar{g} + \bar{f}g} \right)dy.}$$

Equation 15 is similar to the Watson [2] case, but here *f* and *g* depend on *Da*. Having integrated over *y* and *t*, the remaining independent variables for determining *R* are

16 $$R = R\left( {P,\alpha ,Da,Sc} \right).$$

Details of the solution for *R* are given in Additional file 1: Appendix. For validation, this solution reduces to that for a channel without porous media [2] for $Da \to 0$.

### Values of parameters

Results were obtained for the case of periarterial basement membranes and the paraarterial (Virchow-Robin) space within the brain, and for the SSS. For basement membranes, the gap height was taken as 100 nm, which is 75 times smaller than the radius of the smallest arteries (precapillaries ~ 7.5 μm radius), thus the flat plate channel model is justified even for the smallest vessels. The cross section of the basement membrane may be irregular, thus the simplified flat plate channel represents a baseline model from which solutions for more complex geometries may be extended. Molecular diffusivity was taken to be that for amyloid-β, *κ* = 5 × 10^−11^ m^2^/s [46]. This value is for monomers of amyloid-β, which have a size of about 1 nm and thus satisfy the continuum assumption within the channel (oligomers and aggregates of amyloid-β, may be as large as 100 nm, which would violate the continuum model). The density and kinematic viscosity of the suspending fluid taken to be that of water at body temperature, *ρ* = 993 kg/m^3^ and *ν* = 7 × 10^−7^ m^2^/s. The Schmidt number becomes *Sc* = 14,000. The oscillatory frequency was taken as that for the heartbeat, *ω* = 2π rad/s. The Womersley number becomes *α*^*2*^ = 2.24E−8 and the Peclet number *β*^*2*^ = 0.000314.

The pressure gradient driving flow in the basement membrane has not been measured and would be difficult to obtain, given the small sizes involved. Therefore, the approach taken here was to test the ultimate feasibility of transport by oscillatory shear-augmented dispersion by using the largest possible pressure gradient, characterized by cerebral arterial pulse pressure, approximated as 100 mmHg = 13.33 kPa, and a longitudinal distance. This pressure would prevail if the hydraulic resistance (or compliance) across the endothelial layer is small compared to that between the basement membrane and the parenchyma, which near the capillaries comprises pericytes and astrocyte feet. It should be noted that while the intramural pulse pressure in the capillaries has conventionally been thought to be greatly attenuated by flow through the arterioles, evidence suggests that high pressure may persist to the capillaries [47], thus a substantial part of the full pulse pressure may apply to channels beginning at the arteriole/capillary junctions. The pulse pressure in veins is low, thus the potential for driving flow along perivenous channels by venous intramural pressure pulsations is less. Flow might alternatively be driven by pulsations in pressure within the parenchyma if the hydraulic resistance (or compliance) between the intramural space of the vessel (whether artery or vein) and the basement membrane is large compared to that between the basement membrane and the parenchyma. This pulse pressure can be estimated to be that in the CSF, for instance, as measured in the ventricles by a number of investigators (see the following discussion of the SSS). Finally, a longitudinal distance of 0.1 m characterizing the length of cranial vessels gives a maximum nondimensional pressure gradient amplitude of *P* = 1.526.

Permeability of SMC basement membranes has been estimated as 1.432E−18 m^2^ in a rabbit thoracic aorta [48, 49]. Whether cerebral arterial SMC or pial-glial basement membranes are more or less permeable is unknown. Using this value for the current problem makes the Darcy number *Da*^2^ = 1750.

The characteristic thickness of the larger paraarterial space was taken as 10 μm [50, 51]. Taking a cortical arteriole with radius of 11.5 μm [51] as the characteristic vessel size, the gap-to-radius ratio is near unity, thus the flat plate model is a simplification. Again using amyloid-β as the solute, the Schmidt number is *Sc* = 14,000. Using the same heart beat frequency, the Womersley number is *α* = 0.000224 and the Peclet number *β*^*2*^ = 3.14. The driving pressure gradient was assumed the same as for basement membranes, which results in *P* = 152.6. Using a thicker 25 μm channel and a smaller 2.4 Pa/m peak pressure gradient, Bilston et al. [52] nonetheless arrived at a comparable value (*P* = 67) for the paraarterial space of arteries entering the spine. Permeability of the paraarterial space has been estimated as 1.8E−14 m^2^ [53], which makes the Darcy number *Da*^2^ = 1390. If the paraarterial gap is instead comprised by the smaller 100 nm thick pial-gial basement membrane [13, 27], then the parameter values are the same as for the periarterial space.

For the SSS, the gap height was taken as 3 mm (Fig. 3) [34]. This gap prevails along much of the spine, but is considerably larger near the foramen magnum. The perimeter of the SSS (Fig. 3) is only about three times the gap height, thus a flat plate channel model is a simplification. The molecular diffusivity was taken to be that for methotrexate, *κ* = 5.26E−10 m^2^/s ([54] in [55]) (an antimetabolite injected intrathecally to treat cancer), thus the Schmidt number becomes *Sc* = 1330. Using the same heart beat frequency, the Womersley number is *α*^*2*^ = 20.2 and the Peclet number *β*^*2*^ = 26,900. A pressure gradient amplitude of 453 Pa/m was estimated by dividing the pulse pressure of 45.3 Pa [32] by a representative 0.1 m longitudinal distance along the SSS. (A similar pulse pressure (40 Pa) was found in the fourth ventricle in computational fluid dynamics (CFD) simulations of the CSS [38], and this pressure gradient value is comparable to the 525 Pa/m calculated in CFD simulations of flow in the SSS [55, 56]. Other investigations have found higher values, for instance, Williams [57] (pulse pressures of 572 Pa measured in the ventricle and 548 Pa in the lumbar spine in seated subjects) and Heiss et al. [58] (133 Pa in the lumbar spine and 213 Pa in the cervical spine). Differential ventricular to lumbar pulse pressure from Williams [57] (609 Pa), divided by an estimated 61 cm height difference between the two measurement sites gives 1000 Pa/m, roughly double that used in this study.) The nondimensional pressure gradient amplitude becomes *P* = 155.7.

Permeability for the SSS has not been measured, however, permeability in the CSS has been estimated as 2.36 × 10^−8^ m^2^ and porosity as 0.99 [41]. While it could be argued that *k* in the SSS is larger, in the absence of data, this value is used with a channel half-height of 1.5 mm to calculate *Da*^2^ ~ 95.3.

Given the uncertainties regarding permeability throughout the brain and spine, results are presented for several values of *Da*^2^.

### Regimes of flow

Before the results of the analytical solution are shown, an order-of-magnitude analysis of the expected regimes of flow and dispersion is presented in this section. From Eq. 2, the parameters controlling the flow are evident. The pressure gradient drives the flow, and the character of the flow depends on which of the other terms (the unsteady, viscous and Darcy terms) balance it. The coefficient of the viscous term having been normalized to unity and where *ν*_*e*_ ~ *ν*, the ratio of the unsteady term to the viscous term is $\alpha^{2} = \frac{{h^{2} \omega }}{\nu }$ and the ratio of the Darcy term to the viscous term is $Da^{2} = \frac{{h^{2} }}{k}$. These parameters define the following asymptotic regimes of flow: 1. Viscous (Poiseuille) when *α*^*2*^ ≪ 1 and *Da*^*2*^* ≪ 1*, 2. Unsteady when *α*^*2*^ ≫ 1 and *Da*^*2*^*/α*^*2*^ ≪ 1, and 3. Porous (Darcy) when *Da* ^2^ ≫ 1 and *Da*^*2*^/*α*^*2*^* ≫ 1*. The viscous velocity profile is parabolic, with shear from the wall to the center of the channel. For unsteady flow, shear is limited to a boundary layer of dimension $\delta \approx \sqrt {\nu T}$, where *T* is the cycle period. For porous media flow, while shear exists within the media, it is not represented by the continuum model of the Darcy term. In the case of large *Da*^2^, shear is limited to a boundary layer near the wall of thickness $\sqrt k$.

### Regimes of dispersion

These flow regimes impact axial transport by affecting the fraction of the cross section over which displacement gradients create transverse concentration gradients across which diffusion increases axial spread of the molecules. In viscous-dominated oscillatory flow, the Poiseuille velocity profile dictates that the entire cross section participates in enhancing transport. For unsteady flow, the region of transport enhancement is limited to the viscous boundary layer. For porous media flow as modeled by the Darcy term, transport is enhanced only in the Brinkman boundary layer. The effect of transverse diffusion on the enhancement of axial dispersion is influenced in each of these flow regimes by the Peclet number $\beta^{2} = \frac{{h^{2} \omega }}{\kappa }$, which represents the ratio of the time constant for diffusion across the channel to the cycle period. Low *β*^*2*^ corresponds to diffusive transport in which transverse concentration gradients are small throughout the cycle in spite of axial flow, and high *β*^*2*^ corresponds to unsteady dispersion in which transverse diffusion is slow enough that significant transverse concentration gradients are caused by the axial velocity gradients.

Shear-augmented axial transport relative to the maximum advective transport is scaled as [3, 4]

$${\mathscr{D}} = \frac{{w_{rel}^{2} }}{{w_{0}^{2} }}\frac{{t_{c} }}{T}F_{A} ,$$

where *w*_*rel*_ is the characteristic axial velocity of diffusing molecules relative to the average, *t*_*c*_ is the time during which the velocity of the molecules remains correlated and *F*_*A*_ is the fraction of the cross section over which molecules experience relative motion. *w*_*0*_ is the velocity amplitude of the bulk flow, the cyle period scales as *T* ~ 1/*ω* and augmented transport is considered to be additive to molecular diffusion. Maximum axial transport occurs when *w*_*rel*_ = *w*_*0*_, *t*_*c*_ = *T*, and *F*_*A*_ = 1, thus ${\mathscr{D}} = 1$. The augmentation relative to molecular diffusion is found by renormalization

$$R = \frac{{w_{0}^{2} T}}{\kappa }{\mathscr{D}}$$

The maximum augmentation, which occurs for ${\mathscr{D}} = 1$, is $R_{\text{max} } = w_{0}^{2} T/\kappa$. The possible regimes of transport are outlined in the following subsections.

*Viscous flow* (*α*^*2*^* ≪ 1* and *Da*^*2*^* ≪ 1*) *and diffusive dispersion* (*β*^*2*^* ≪ 1*)—For this case, the relative velocity scales with that of the bulk flow *w*_*rel*_ ~ *w*_*0*_, the correlation time scales with the time for diffusion across the cross section *t*_*c*_ ~ *h*^*2*^/κ, and the whole cross section is involved *F*_*A*_ ~ 1, thus

$${\mathscr{D}} \sim \beta^{2} .$$

To estimate *R*, the characteristic velocity scales as $w_{0} \sim h\omega P$, thus

$$R{\sim }P^{2} \beta^{4} .$$

Maximum enhancement is achieved by reducing lateral dispersion such that *t*_*c*_ = *T*

$$R_{\text{max} } {\sim }P^{2} \beta^{2} .$$

*Viscous flow* (*α*^*2*^* ≪ 1* and *Da*^*2*^/*α*^*2*^* ≪ 1*) *and unsteady dispersion* (*β*^*2*^* ≫ 1*)—For this case, the relative velocity is limited to the velocity difference across a characteristic diffusion distance $w_{rel} \sim w_{0} \sqrt {\kappa T} /h$, the correlation time is limited to the cycle period *t*_*c*_ ~ *T*, while the whole cross section is still involved *F*_*A*_ ~ 1, thus

$${\mathscr{D}} \sim \beta^{ - 2} \;{\text{and}}\;R \approx P^{2} .$$

*Since R*_*max*_ always requires *t*_*c*_ ~ *T* and *F*_*A*_ ~ 1, it depends only on *w*_*0*_, and thus on the type of flow. For this case, *R*_*max*_ is achieved by increasing lateral dispersion such that *w*_*rel*_ = *w*_*0*_

$$R_{\text{max} } {\sim }P^{2} \beta^{2} .$$

*Unsteady flow* (*α*^*2*^* ≫ 1* and *Da*^*2*^/*α*^*2*^* ≪ 1*) *and unsteady dispersion* (*β*^*2*^* ≫ 1*)—For large Schmidt number, the molecular diffusion distance is smaller than the viscous diffusion distance. The relative velocity occurs over the smaller distance, while the maximum velocity difference in exhibited across the viscous boundary layer $w_{rel} \sim w_{0} \sqrt {\kappa T} /\sqrt {\nu T}$. The correlation time is limited to the cycle period *t*_*c*_ ~ *T*, and the fraction of the cross section with velocity gradients is that of the oscillatory boundary layer $F_{A} \sim \sqrt {\nu T} /h$, thus

$${\mathscr{D}}\sim \beta^{ - 1} Sc^{ - 1/2} .$$

The characteristic velocity scales as $w_{0} \sim \frac{\nu }{h}P$, thus

$$R{\sim }P^{2} \alpha^{ - 3} .$$

Maximum enhancement is reached by increasing lateral dispersion such that *w*_*rel*_ = *w*_*0*_ and adding velocity gradients in the core flow such that *F*_*A*_ = 1

$$R_{\text{max} } {\sim }P^{2} \alpha^{ - 2} Sc.$$

For small Schmidt number (which is not characteristic of the problems addressed in this paper), the molecular diffusion distance is larger than viscous diffusion distance. The relative velocity is, therefore, that over the whole viscous boundary layer, making $w_{rel} \sim w_{0}$. The correlation time scales with the time for diffusion across the viscous boundary layer *t*_*c*_ ~ *νT*/κ, and the fraction of the cross section with velocity gradients is that of the oscillatory boundary layer $F_{A} \sim \sqrt {\nu T} /h$, thus

$${\mathscr{D}}\sim \alpha^{ - 1} Sc \quad {\text{and}}\;R\sim P^{2} \alpha^{ - 3} Sc^{2} .$$

Maximum enhancement is achieved by decreasing lateral dispersion such that *t*_*c*_ = *T* and adding velocity gradients in the core flow such that *F*_*A*_ = 1

$$R_{\text{max} } {\sim }P^{2} \alpha^{ - 2} Sc.$$

*Porous flow* (*Da*^*2*^* ≫ 1* and *Da*^*2*^/*α*^*2*^* ≫ 1*) *and diffusive dispersion* (*Da*^*2*^*/β*^*2*^* ≫ 1*)—For large $\frac{{Da^{2} }}{{\alpha^{2} }} = \frac{\nu }{k\omega }$, the Brinkman layer is smaller than the unsteady viscous boundary layer, thus F_A_ ~ $\sqrt k /h$. For large $\frac{{Da^{2} }}{{\beta^{2} }} = \frac{\kappa }{k\omega }$, the molecular diffusion distance during one cycle is greater than the Brinkman layer. The relative velocity is, therefore, that over the whole Brinkman layer $w_{rel} \sim w_{0}$. The correlation time is the time for diffusion across the Brinkman layer *t*_*c*_ ~ *k*/κ, so

$${\mathscr{D}}\sim \beta^{2} Da^{ - 3} .$$

The characteristic velocity scales as $w_{0} \sim \frac{k\omega }{h}P$, thus

$$R{\sim }P^{2} \beta^{4} Da^{ - 7} .$$

Maximum enhancement is achieved by decreasing lateral dispersion such that *t*_*c*_ = *T* and adding velocity gradients in the core flow such that *F*_*A*_ = 1

$$R_{\text{max} } {\sim }P^{2} \beta^{2} Da^{ - 2} .$$

*Porous flow* (*Da*^*2*^* ≫ 1 and Da*^*2*^/*α*^*2*^* ≫ 1*) *and unsteady dispersion* (*Da*^*2*^*/β*^*2*^* ≪ 1*)—For small $\frac{{Da^{2} }}{{\beta^{2} }} = \frac{\kappa }{k\omega }$, the molecular diffusion distance during one cycle is smaller than the Brinkman layer. The relative velocity occurs over the smaller distance, so $w_{rel} \sim w_{0} \sqrt {\kappa T} /\sqrt k$. The correlation time is the cycle period *t*_*c*_ ~ *T*, and

$${\mathscr{D}}\sim \beta^{ - 2} Da \quad {\text{and}}\;R\sim P^{2} Da^{ - 3} .$$

Maximum enhancement is achieved by increasing lateral dispersion such that *w*_*rel*_ = *w*_*0*_ and adding velocity gradients in the core flow such that *F*_*A*_ = 1

$$R_{\text{max} } {\sim }P^{2} \beta^{2} Da^{ - 2} .$$

## Results

### Velocity

**Fig. 4:**
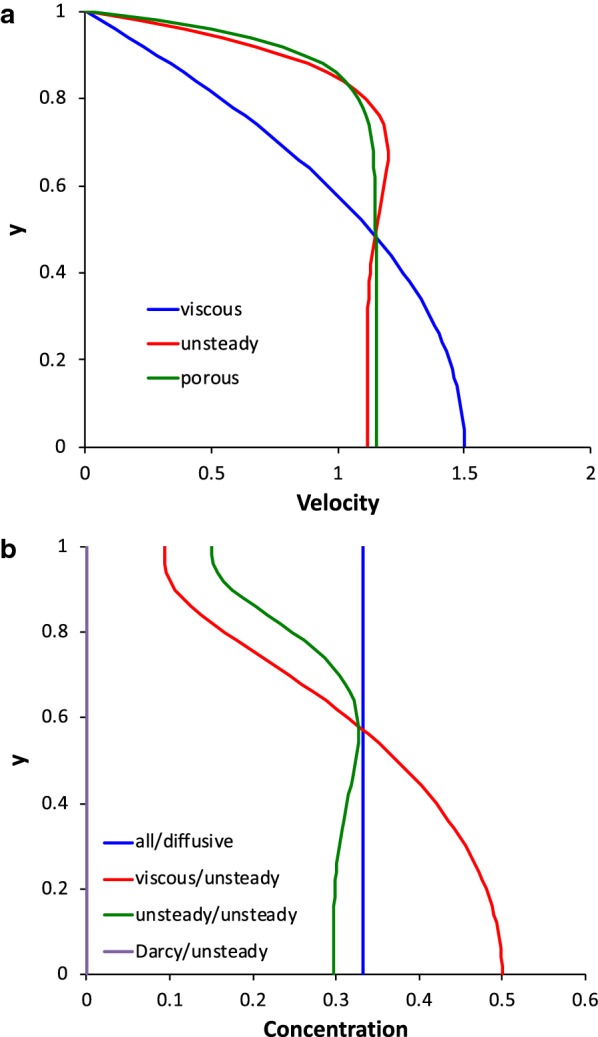
**a** Characteristic dimensionless velocity (relative to the mean velocity) profiles versus dimensionless distance from the center of the channel (relative to the channel half height) for the three regimes of flow. The viscous profile is parabolic (Poiseuille). The porous profile is flattened by the resistance to flow through the porous media. The unsteady profile exhibits a peak between the core and the boundary layer due to fluid inertia. **b** Characteristic dimensionless concentration profiles versus dimensionless distance from the center of the channel for the regimes of dispersion. The profiles mirror those of velocity, except for the no-flux boundary condition at the wall. In the legend, the flow regime is given before the slash and the dispersion regime after the slash. The unsteady curves are shown for Womersley number *α*^*2*^ = 100, and the porous curves are shown for Darcy number *Da*^2^ = 200

Characteristic velocity profiles from the analytical solution for the three cases are shown in Fig. 4a. When the viscous term dominates, the profile is parabolic (Poiseuille) and the peak velocity is 1.5 times the average. For unsteady, inertia-dominated flow, a core of uniform velocity develops, with a surrounding intermediate layer that can have higher velocity as shown in Fig. 4a, and a viscous boundary layer near the wall (shown for *α*^*2*^ = 100). Due to the fluid inertia, the velocities of the core and intermediate layer respond out of phase to the pressure gradient, with the lag being greatest for the core and least near the wall, which creates the inflection in the velocity profile. When the flow is dominated by resistance through the porous media, the core has a constant velocity, but a no-slip boundary condition still applies at the wall (shown for *Da*^2^ = 200). The resistance effect dominates that of fluid inertia, thus velocity across the whole cross section responds in phase with pressure and no inflection occurs.

### Concentration

Although there are six regimes of dispersion, two (diffusive and unsteady) for each of the three flow regimes, only four unique concentration profiles occur. When the transport is diffusive, regardless of the velocity regime, rapid diffusion across the cross section causes the concentration to be uniform (Fig. 4b). The three remaining regimes are unsteady dispersion in viscous, unsteady and porous flow. For each of these regimes, diffusion is weak, thus the concentration profile is driven by the velocity gradients. The concentration profiles mirror the velocity profiles (Fig. 4a) except near the wall, where the no-flux boundary condition for concentration dictates a concentration gradient of zero.

### Enhancement of axial dispersion

**Fig. 5:**
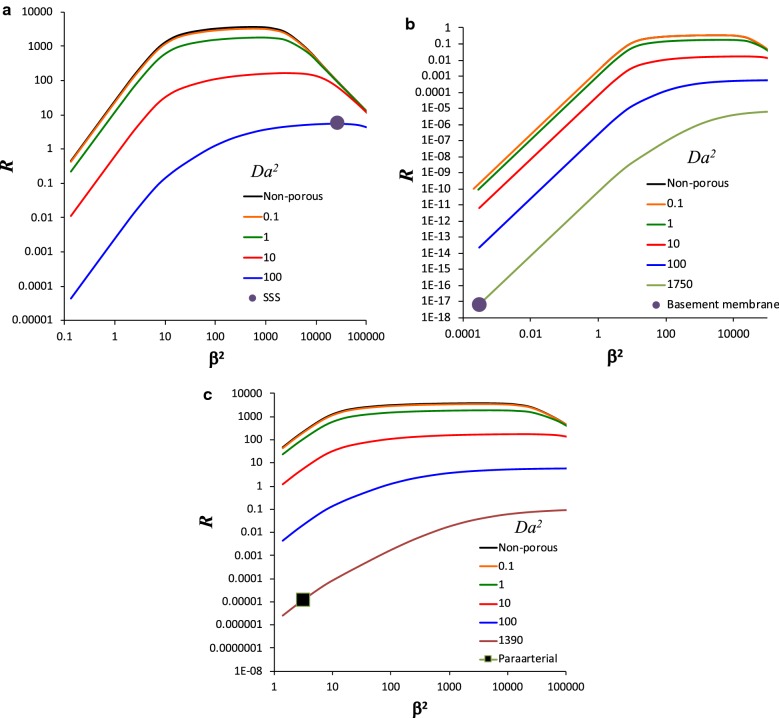
**a** Dispersion enhancement *R* for Schmidt number *Sc* = 1330 and dimensionless pressure gradient *P* = 155.7. Enhancement is significant (> 1) in the SSS, the conditions for which are estimated by the large dot (Peclet number *β*^*2*^ = 26,900 and Darcy number *Da*^*2*^ = 95.3). **b** Dispersion enhancement for *Sc* = 14,000 and *P* = 1.526. Enhancement is very small for cerebrovascular basement membranes, as shown by the large dot (*β*^*2*^ = 0.00314 and *Da*^2^ = 1390). **c** Dispersion enhancement for *Sc* = 14,000 and *P* = 152.6. Enhancement is small in the larger paraarterial space, as shown by the large dot (*β*^*2*^ = 3.14 and *Da*^2^ = 1750)

For *Sc* = 1330 and *P* = 155.7, characteristic of methotrexate in the SSS, enhancement of axial dispersion *R* reaches a maximum of about 3500 over a range of α^2^ from 0.0001 to 100, which corresponds to *β*^*2*^ from 0.133 to 1.33E+5 (Fig. 5a). The regimes of flow and dispersion are evident from the curves. For low *Da*^2^, *R* increases with increasing *β*^*2*^ in the viscous flow/diffusive dispersion regime to a level of *R* ~ 3000 at which the dispersion begins to transition to unsteady at around *β*^*2*^ ~ 1. *R* then increases slightly with increasing *β*^*2*^ in the viscous flow/unsteady dispersion regime to another transition at about α^2^ ~ 1 (*β*^*2*^ = 1330). Beyond this transition, the flow becomes unsteady while the dispersion remains unsteady, and *R* decreases. The porous media decreases *R* beginning at about *Da*^2^ = 1, and also softens the transition between steady and unsteady dispersion, as well as between steady and unsteady flow (most evident in the *Da*^2^ = 100 curve), because both the viscous and unsteady boundary layers are both small. As predicted by the order of magnitude scaling, *R* increases proportional to *β*^*4*^ for diffusive dispersion, is relatively insensitive to *β* for viscous flow/unsteady dispersion and for porous flow/unsteady dispersion, and decreases proportional to *β*^−*3*^ for unsteady flow/unsteady dispersion. (The curve for *Da*^*2*^ = 100 does not transition to unsteady flow, which requires *Da*^*2*^/α^*2*^* ≪ 1,* within the bounds of the plot. This parameter only reaches *Da*^*2*^/α^*2*^=* 1* for the maximum value of *β*^*2*^ = 1.33E+5.) The nearly identical curves for *Da*^*2*^ = 0.1 and the non-porous case Watson [2] show that the effect of the porous media is small for values of $Da^{2} \le 0.1$. The convergence of all the curves for large *β*^*2*^ regardless of *Da*^2^ indicates transition to the unsteady flow regime, where the viscous boundary layer is smaller than the Brinkman layer.

For *Sc* = 14,000 and *P* = 1.526, characteristic of amyloid-β in cerebrovascular basement membranes, enhancement of axial dispersion *R* is minimal, rising only to about 0.3 over a range of *α*^*2*^ from 1E−8 to 10, which with the higher *Sc* corresponds to *β*^*2*^ from 0.00014 to 1.4E+5 (Fig. 5b). The dispersion transitions from diffusive to unsteady at the same *β*^*2*^ ~ 1, however the peak *R* is much lower. The flow again transitions from viscous to unsteady around *α*^*2*^ ~ 1, though due to the higher *Sc*, this transition appears in Fig. 5b at *β*^*2*^ ~ 14,000. The same flow and dispersion-dependent rates of increase and decrease of *R* are exhibited, and increasing *Da*^2^ decreases transport and softens the transitions. Similar agreement of the behavior of *R* with the scaling predicted by order of magnitude analysis is evident.

For *Sc* = 14,000 and *P* = 152.6, characteristic of amyloid-β in the larger (10 μm) paraarterial space, enhancement of axial dispersion *R* of nearly 4000 is possible over a range of *α*^*2*^ from 0.0001 to 1E+5, which corresponds to *β*^*2*^ from 1.4 to 1.4E+9 (Fig. 5c). Over this range, the flow and dispersion are both mostly unsteady, with the transition to diffusive to unsteady dispersion beginning immediately at the low *β*^*2*^ end of the curves for low *Da*^2^. The flow again transitions from viscous to unsteady at *β*^*2*^ ~ 14,000 (α^2^ ~ 1).

Having solved the general problem, we turn to the estimated conditions specific to dispersion in the spine and in cerebrovascular basement membranes. For the SSS, the Womersley, Peclet and Darcy numbers are *α*^*2*^ ~ 20.2, *β*^*2*^ ~ 26,900 and *Da*^2^ ~ 95.3, respectively. The resulting dispersion enhancement is *R* = 5.80 (Fig. 5a). It can be seen in Fig. 5a that if the permeability were large enough that the effect of the porous media were insignificant (*Da*^2^ = 0), the enhancement would be *R* = 91.8.

For cerebrovascular basement membranes, the Womersley and Peclet numbers are *α*^*2*^ ~ 2.24E−8 and *β*^*2*^ ~ 0.000314, respectively. For an estimated Darcy number of *Da*^2^ = 1750, dispersion enhancement is *R* = 6.38E−18 (Fig. 5b). For a nonporous media, enhancement increases to *R* = 2.42E−10.

For the 100 times larger version of the paraarterial space, the Womersley and Peclet numbers increase to *α*^*2*^ ~ 0.000224 and *β*^*2*^ ~ 3.14, respectively. For an estimated Darcy number of *Da*^2^ = 1390, dispersion enhancement is *R* = 1.178E−5 (Fig. 5c). For nonporous media, enhancement increases to *R* = 220.

## Discussion

Using the continuum model of oscillatory flow in porous media, shear-augmented dispersion has a significant effect on transport of methotrexate in the SSS, but amyloid-β is about eighteen orders of magnitude away from significance for cerebrovascular basement membranes and five orders of magnitude for the larger pararterial space. The order of magnitude estimate of maximum transport enhancement (“Regimes of dispersion” section), however, implicitly incorporates phenomena that alter transverse mixing without changing the oscillatory longitudinal velocity amplitude and zero mean flow. Two such effects, local effects on axial velocity and secondary transverse flow, are discussed in the following subsections.

### Local velocity fluctuations

**Fig. 6:**
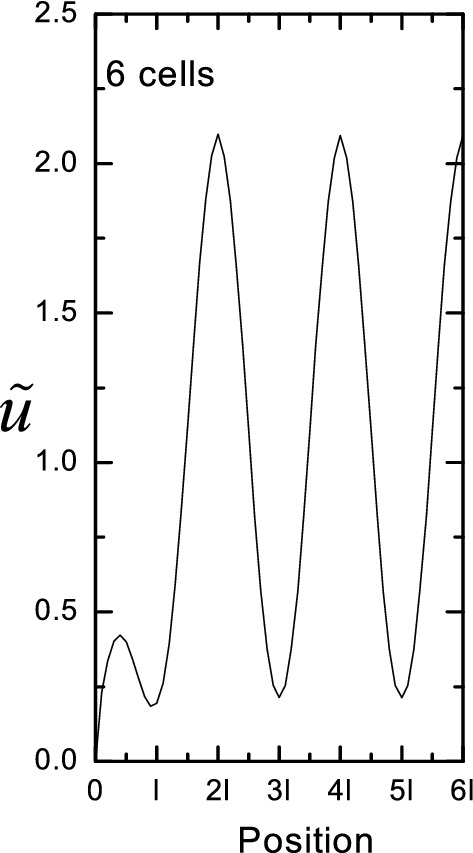
Example superficial velocity $\tilde{u}$ profile within a square array of cylinders. Position is from a flat wall on the left to the center of the channel on the right. 2*l* is the spacing between cylinders. The velocity gradients created by the high velocity in the gap between cylinders and the low velocity downstream of cylinders provides the potential for enhanced dispersion. (From [77])

The no-slip boundary condition brings axial velocity to zero where the fluid contacts the media, and axial velocity is locally accelerated in passages through the solid material. Both of these effects increase shear and concentration gradients locally, which can be expected to increase axial dispersion. An example superficial velocity profile is shown in Fig. 6, in which spatial fluctuations in velocity remain downstream of a square array of cylinders between flat plates. The fluid in the high velocity regions between cylinders carries molecules forward, creating local transverse concentration gradients that do not exist in the Darcy model of porous media flow. If the regime of transport is not already diffusive, then the added transverse transport increases axial dispersion.

### Secondary flow

Transverse flow in porous media is characterized by tortuosity, which is a ratio of the distance along a streamline to the distance between its end points. The effect of tortuosity on dispersion may be minimal if the tortuous channels do not communicate with adjacent channels. However, if mixing occurs between channels with different concentration, then the impact on axial dispersion can be large in regimes of dispersion in which transverse diffusion is weak. Simulations of flow and dispersion in unit cells representing regular, periodic geometries of simplified porous media have demonstrated enhancements of longitudinal dispersion by as much as four orders of magnitude (in a two-dimensional, hexagonal array of circular cylinders [59]).

Oscillatory annular (nonporous) flow with axial velocity that has phase differences (axial velocity is forward for half the annulus while the other half is reverse) and transverse secondary flow also provides a model of this effect [4]. Axial dispersion in this model parallels that in flows without secondary flow in that a peak in enhancement occurs in the transition between regimes of low and high transverse transport. In this case, transverse transport occurs not only by diffusion, but also by advection. The peak occurs were *t*_*s*_/*T* ~ 1, where *t*_*s*_ is the secondary flow time. Axial dispersion increases as *t*_*s*_/*T* approaches unity from either side, but in addition, convective resonance occurs at *t*_*s*_/*T* ~ 1, where secondary flow carries molecules a half circuit around the annulus in half a cycle (from a region of forward velocity to a region that a half cycle later also has forward velocity). This keeps the molecule advecting in a consistent direction, in spite of the reversal of axial flow, increasing axial dispersion by up to an additional two orders of magnitude. Similar, but weaker, resonance occurs when the secondary displacement during a cycle is an integer multiple of the annulus circumference.

### Maximum enhancement

As outlined in “Regimes of dispersion” section, maximum enhancement $R_{\text{max} } = w_{0}^{2} T/\kappa$ occurs when the relative velocity of particles scales with the characteristic velocity of the fluid, the particles move with that relative velocity for a whole cycle and the entire cross section is involved. For the unsteady dispersion in the SSS, increased lateral mixing, for instance by local velocity fluctuations or secondary flow (“Local velocity fluctuations and secondary flow” sections), is required to achieve this condition, and enhancement could be increased from *R* = 5.80 to *R*_*max*_ = 1.60E+6. The model predicts that the characteristic time $t\sim L^{2} /\left[ {\kappa \left( {1 + R} \right)} \right]$ for methotrexate to be transported along a *L* = 0.7 m long spinal canal decreases from 4.3 year to 9.7 min, which is clinically useful. The corresponding characteristic transport speed $v\sim \left[ {\kappa \left( {1 + R} \right)} \right]/L$ increases from 5.1E−6 mm/s to 1.2 mm/s.

For basement membranes, reduced lateral dispersion increases enhancement from *R* = 6.38E−18 to *R*_*max*_ = 0.000730. Characteristic transport time for amyloid-β on a 0.1 m long path along the cerebral arterial tree is about 6.3 year in either case. This time is much too long to explain observed transport of solutes [12], therefore, some other mechanism must be responsible.

For a 10 μm paraarterial space, reduced lateral dispersion increases enhancement from *R* = 1.178E−5 to *R*_*max*_ = 73,200, which produces a characteristic transport time for amyloid-β along the cerebral arterial tree of 45 min. While promising, this time may be deceiving, because the gap is thought to be much smaller around precapillaries, which would lead to enhancement there that is more similar to that of basement membranes.

### Comparison with previous work

The only previous model of perivascular or paravascular transport of which we are aware is that of Asgari et al. [51]. Their model is very different, representing a 10 μm thick paravascular space filled with porous media surrounding short (150–250 μm) sections of cortical arterioles (23 μm diameter). Pulsatile motion of the inner wall of the space was imposed, while zero pressure, uniform velocity and constant concentration boundary conditions were set at the ends of the segment. The resulting pulsatile, squeeze flow and unsteady dispersion produced *R* ~ 1. This enhancement is greater than that found here for the Darcy–Brinkman result (*R* = 1.178E−5), which may be attributable to the greater transverse flow, but still produces a long characteristic time of *t* ~ 3 year for transport of a solute with *κ* = 5E−11 m^2^/s along a 0.1 m path.

Stockman [60] modeled the SSS as an elliptical annulus and compared axial transport for a non-porous channel and a channel with nerve bundles converging at the dural surface and trabeculae with random orientation. Lattice-Boltzmann simulations with *α* = 11 (larger than the *α* = 4.49 assumed in this paper) and 10 < *Sc* < 100 (smaller than the *Sc* = 1330 for methotrexate used in this paper) predicted enhancements of approximately 0.5 for the non-porous channel and 2.5 for the channel with nerve bundles and trabeculae. The differences in parameter values from the present work notwithstanding, the roughly 5-fold increase in effective diffusivity by porous media found by Stockman demonstrates its potential to increase transverse mixing and, therefore, longitudinal transport.

A fivefold transport enhancement by pulsatile flow was reported in a simplified model of the SSS without porous media [61]. This value is lower than the 11-fold value calculated using the parameters of these experiments for the Watson limit of the Darcy–Brinkman model. One difference between their experiments and the Watson model is that the annular channel height to outer radius ratio was perhaps too large at 0.12 to fit the flat plate channel assumption of the Watson solution. In addition, the pulsatile flow waveform was more complex than the simple oscillatory flow of the Watson solution.

A greater reduction in peak drug concentration was found due to doubling the tidal volume than by doubling the frequency in a patient-specific geometry without porous media [62]. This result is in qualitative agreement with the Watson solution, which predicts that *R* is proportional to the square of tidal volume and, in the limit of large Womersley number, is approximately proportional to frequency.

While Tangen et al. [63] did not quantify effective diffusivity, they reported more rapid spread of drugs caused by local mixing around nerve roots and trabeculae. Interestingly, dispersion was not significantly influenced by molecular diffusivity for variations around a baseline of 2.1E−10 m^2^/s for bupivacaine. This finding suggests *R* in their simulations was roughly proportional to *β*^−*2*^ (since molecular diffusivity is in the denominator of *β*^*2*^). While the molecular diffusivity for bupivacaine is lower than for the methotrexate used in this paper, the flow and dispersion both remain unsteady. In Fig. 5a, it is evident for the Darcy–Brinkman model that the enhancement in the unsteady flow/unsteady dispersion regime transitions from *R* α *β*^−*3*^ to *R* ~ constant in the range 1 < *Da*^2^ < 100, suggesting that the effective Darcy number of their flow was in this range.

Tangen et al. [64] studied a number of parameters associated with drug injection, pulsatility and drug reaction rate in two subject-specific geometries with nerve roots. While again not quantifying effective diffusivity, they noted transport speed for an injection into the lumbar spine in in vitro and computer models in the range of 0.013 mm/s. Pizzichelli et al. [65] and Haga et al. [66] investigated the effect of catheter position and orientation on intrathecal isobaric drug dispersion within the cervical spine with anatomically realistic nerve roots. In both of these studies they found local solute dispersion to be sensitive to catheter position, orientation and anatomy (nerve roots). However, the highly computationally expensive simulations were carried out for a relatively short time scale and therefore it was not possible to draw conclusions about global solute distribution times.

### Limitations

The 2D channel approximation is appropriate for basement membranes, but dura-radius-to-gap ratio for the SSS is only about 3 (“Values of parameters” section), making the 2D analytical solution questionable. The order-of-magnitude scaling for maximum enhancement, however, depends on channel shape only through the characteristic velocity w_0_. For Poiseuille flow, the ratio of peak velocity in an annulus to that in a 2D channel scales with $18\left[ {1 - \lambda^{2} \left( {1 - \ln \lambda^{2} } \right)} \right]$, where $\lambda^{2} = \left( {1 - K^{2} } \right)/\left[ {2\ln \left( {1/K} \right)} \right]$ and $K = 2/3$ for the SSS, which results in a velocity in the annulus that is 1.004 times larger and enhancement $R_{\text{max} } \propto w_{0}^{2}$ that is 1.009 larger. Therefore, this limitation is not very significant.

In addition to lacking local effects (“Local velocity fluctuations” section) and secondary flow (“Secondary flow” section), the analytical solution does not apply for short times after injection of a bolus. Consideration of short times may result in other opportunities for improving rostral transport, for instance, by injecting at a particular time during the cycle (i.e., during maximum caudal displacement of the CSF fluid), by the orientation of the injection catheter, by the velocity of the injection and by following the injection with a bolus of clear fluid to push the solute upward.

Periodic motion of the channel walls, as well as geometries more complex than the planar walls of the current model, also promote transverse flows that may enhance transverse mixing and axial transport. In particular, streaming effects (reviewed by Riley [67]) can occur in flows with relevance to the SSS, for instance, in the entrance region of oscillatory flow in a rigid tube [68], in a long, but finite, parallel-plate channel with oscillating walls [69], in an elastic tube [70], in a tapered channel [71], in an elliptical tube with oscillating walls [72], and in a closed-end, compliant, eccentric circular annulus [73] and an elliptical annulus [74] modeling the SSS. In both models of the SSS, streaming velocities of 0.1–0.3 mm/s were obtained, which provide characteristic transport times for a 0.7 m spinal canal of 0.7–2 h.

## Conclusions

The Darcy–Brinkman model, which represents the porous media flow as a continuum, predicts a decrease in axial dispersion as the Darcy term increases, across all regimes of viscous and porous-media flow and diffusive and unsteady dispersion, but not for unsteady flow and unsteady dispersion. For CSF flow in the SSS, which is estimated to be in the transition zone between porous-media and unsteady flow, the Darcy–Brinkman model predicts substantial increases in axial transport due to shear-augmented dispersion, so long as the effect of the continuum porous media is not too great. However, for cerebrovascular basement membranes, which is estimated to exhibit quasi-steady flow and dispersion, augmentation is minimal regardless of whether the porous media is included or not.

Order of magnitude estimates with altered transverse dispersion due to local effects of the porous media predict greater enhancement of transport. In the SSS, increased lateral transport leads to an enhancement by as much as six orders of magnitude and a characteristic transport time along the spinal canal of about 10 min and characteristic transport speed of 1.2 mm/s. This time is 2–6 times faster than observed in in vitro experiments, suggesting that dispersion might be improved through optimal selection of operating parameters. This speed is 4–12 times faster than simulations excluding diffusion [73, 74], suggesting that shear-augmented dispersion might have therapeutic value for increasing transport rates.

According to the relationship $R\sim P^{2} Da^{ - 3}$ for porous flow and unsteady dispersion (see “Regimes of dispersion” section), greater transport approaching *R*_*max*_ in the SSS could be promoted by increasing *P*, for instance, by increasing the pressure gradient amplitude. *R* is also increased by decreasing frequency, since $P^{2} \propto \omega^{ - 2}$. Respiration has been shown to affect SSS flow [75], so deep inspiration and expiration may be effective in providing an elevated pressure gradient at low frequency. While the fluid properties may be unalterable, the spine is flexible. Thus, increased curvature of the SSS might increase secondary flow and transverse mixing, thereby shifting enhancement of longitudinal transport toward R_max_.

In a 10 μm paraarterial space, enhancement has the potential to be significant, thus glymphatic transport to the parenchyma is not disproven. However, the low pulse pressure in veins makes glymphatic transport out of the parenchyma via paravenous spaces unlikely. In cerebrovascular basement membranes, the small estimated amplitude of motion limits the enhancement of transport. Even with lateral dispersion reduced to match it to the cycle period, maximum enhancement is insignificant.

The lack of significant shear-augmented dispersion in basement membranes means that within the bounds of the channel flow model, tracer transport must be explained by bulk flow, since this is the only other available mechanism in this simplified model. Peristalsis is a plausible cause of forward flow in periarterial and paraarterial channels, but perhaps not in perivenous channels since blood pressure pulsations are low in veins. Three potential mechanisms for retrograde flow in periarterial basement membranes have been described (see “Perivascular and paravascular flow and transport” section), but not verified. Therefore, further work remains to test these hypotheses and to explain the mechanisms of solute movement in these channels.

Finally, an overarching need is to reduce uncertainty regarding the anatomy and fluid dynamic parameters characterizing the perivascular and paravascular spaces, which may vary among species and between genders [76].

## List of symbols

*c*: concentration; *c*_*0*_: characteristic concentration; $Da^{2} = \frac{{h^{2} \nu }}{{k\nu_{e} }}$: square of the Darcy number; *h*: channel half height; *k*: permeability; $\tilde{p}$: pressure; $p = \frac{{\tilde{p}}}{{\rho \omega \nu_{e} }}$ dimensionless pressure; $P = \frac{{\partial \tilde{p}/\partial \tilde{x}}}{{\rho \omega \nu_{e} /h}}$: dimensionless pressure gradient; *R*: dispersion enhancement relative to molecular diffusion; *R*_*max*_: maximum dispersion enhancement; $Sc = \nu /\kappa$: Schmidt number; $\tilde{t}$: time; $t = \omega \tilde{t}$: dimensionless time; $\tilde{u}_{s}$: superficial axial velocity; $u = \tilde{u}_{s} /h\omega$: dimensionless superficial velocity.

### Variables

$\tilde{x}$: axial coordinate; $x = \tilde{x}/h$: dimensionless axial coordinate; $\tilde{y}$: transverse coordinate; $y = \tilde{y}/h$ dimensionless transverse coordinate.

### Greek symbols

$\alpha^{2} = \frac{{h^{2} \omega }}{{\nu_{e} }}$: square of the Stokes (Womersley) number; $\beta^{2} = \frac{{h^{2} \omega }}{\kappa } = \alpha^{2} Sc$: oscillatory Peclet number; $\theta = \frac{c}{{c_{0} }}$: dimensionless concentration; *κ*: molecular diffusivity; *ν*: kinematic viscosity of the fluid; *ν*_*e*_: effective kinematic viscosity for flow in the porous medium; *ρ*: fluid density; *ω*: frequency.

## Additional file

**Additional file 1.** Appendix.

## Abbreviations

CFD: computational fluid dynamics
CNS: central nervous system
CSF: cerebrospinal fluid
CSS: cortical subarachnoid space
IPAD: intramural periarterial drainage
ISF: interstitial fluid
MCA: middle cerebral artery
SMC: smooth muscle cell
SSS: spinal subarachnoid space
VRS: Virchow-Robin space

## Footnotes

1 The Womersley number has the same form as the earlier-defined Stokes number used in this paper (see definition after Eq. 2).
