# Supplementary material for: Dispersion in porous media in oscillatory flow between flat plates: applications to intrathecal, periarterial and paraarterial solute transport in the central nervous system
Source: Fluids Barriers CNS. 2019 May 6;16:13. doi: 10.1186/s12987-019-0132-y (PMC6512764; doi:10.1186/s12987-019-0132-y)
Supplement: Supplementary file 1 — Additional file 1. Appendix. [file 12987_2019_132_MOESM1_ESM.doc]

**Appendix**

To solve using complex conjugates, the variables are separated into real and imaginary parts. First for *f*

$f=M_{f}+\text{iN}_{f}$ and ,

where

and , and

and ,

where

and , and

and .

Similarly for *g*

$g=M_{g}+\text{iN}_{g}$ and ,

where

and ,

and ,

and ,

and ,

and ,

and ,

and ,

and ,

and ,

$M_{m}=M_{B}m-N_{B}n$ and $N_{m}=M_{B}n+N_{B}m$,

and ,

and ,

and , and

and .

For the integrand of the shear augmentation integral (equation 15)

,

where *C_i_* are constants and *I_i_* are variables in *y,*

,

,

,

,

,

,

,

,

, and

.

The enhancement factor (equation 15) becomes

,

where the definite integrals are

,

,

,

,

,

,

,

,

and

.
